# Interaction of an Iris Implantation Cyst with Pseudophakic Bullous Keratopathy: A Case Report †

**DOI:** 10.3390/reports9010080

**Published:** 2026-03-10

**Authors:** Răzvan-Geo Antemie, Raluca-Margit Szilveszter, Costina Stafie, Sorin Simion Macarie

**Affiliations:** 1 Department of Physiology, Faculty of Medicine, “Iuliu Haţieganu” University of Medicine and Pharmacy, 400006 Cluj-Napoca, Romania; antemie_razvan_geo@elearn.umfcluj.ro; 2 Department of Pathology, Faculty of Medicine, “Iuliu Haţieganu” University of Medicine and Pharmacy, 400340 Cluj-Napoca, Romania; 3 Faculty of Medicine, “Iuliu Haţieganu” University of Medicine and Pharmacy, 400006 Cluj-Napoca, Romania; stafie.costina@elearn.umfcluj.ro; 4 Department of Ophthalmology, Faculty of Medicine, “Iuliu Haţieganu” University of Medicine and Pharmacy, 400006 Cluj-Napoca, Romania; sorin.macarie@umfcluj.ro

**Keywords:** iris cyst, pseudophakic bullous keratopathy, cornea, phacoemulsification, epithelial downgrowth

## Abstract

**Background and Clinical Significance**: Currently, the only effective treatment for cataracts is surgery. The most commonly employed method is phacoemulsification, a well-established procedure that uses ultrasound energy to fragment the lens, allowing for easier removal. Potential postoperative complications range from mild to severe corneal edema (pseudophakic bullous keratopathy), which may be caused by intraoperative endothelial cell damage, to the rare formation of iris cysts. **Case Presentation**: In this paper, we report the case of a patient who underwent cataract surgery in both eyes, where iris incarceration occurred during the surgical procedure, resulting in corneal edema and an iris cyst, both in the left eye. Secondary iris cysts are uncommon following phacoemulsification, with only a few cases reported in the recent literature. The cyst’s impact on corneal edema was unexpected, making this case particularly noteworthy and emphasizing the complexity of cataract surgery and its postoperative complications. **Conclusions**: This case illustrates the unique interaction between two infrequent complications of cataract surgery.

## 1. Introduction and Clinical Significance

Pseudophakic bullous keratopathy (PBK) is a relatively rare complication following modern cataract surgery, yet it is a significant cause of visual morbidity. When substantial intraoperative surgical trauma to the corneal endothelium coincides with a low count of these cells, corneal decompensation and edema may develop over time. The current incidence in the USA is around 0.1% [1].

Iris masses observed during a slit-lamp examination raise the suspicion of iris or ciliary body tumors, but in most cases, these are benign, and frequently they are cysts [2]. As it is important to differentiate iris cysts from melanomas, the group led by Shields [3] intended to differentiate them using criteria such as morphology, mass location, or the nature of iris elevation. His classification of iris cysts divides them into primary and secondary cysts, depending on tissue origin [4]. Primary cysts are of epithelial origin, have thin, regular walls, and are less than 3 mm in size.

The origin of secondary cysts can range from conjunctival or corneal epithelium to eyelid skin epithelial cells, or even from tumor metastasis arising from lung carcinoma [5]. Chronic use of miotics has also been linked to the development of these cysts, although the most common causes are penetrating trauma or surgical interventions [6]. Epithelial downgrowth (ED), a rare but serious condition, is primarily associated with vitreous loss, capsule rupture, multiple surgeries, delayed wound healing, and iris incarceration, particularly in extracapsular cataract surgery. If left untreated, ED can lead to irreversible vision loss [7]. Epithelial cells from the conjunctiva or cornea are introduced into the globe through surgical or traumatic breaches, and upon encountering various intraocular structures, they begin to proliferate. ED can manifest in three forms: an opaque sheet over the cornea, iris, and angle structures, an epithelial anterior chamber (AC) clear cyst, or, rarely, as a pearl-white solid cyst, also called an iris pearl tumor [1,8,9]. The sheet-like form has the worst prognosis out of the three. Pearl cysts are commonly associated with cilia entering the AC. The cyst’s cavity often contains clear fluid or cellular debris from epithelial desquamation, and, though non-inflammatory, this fluid may exert pressure on nearby ocular structures, depending on the cyst’s size. Histopathological examinations reveal that these cysts are typically lined by stratified squamous epithelium resembling either conjunctival or corneal epithelium, which may be non-keratinized or partially keratinized, with variable thickness and supported by a distinct basement membrane. Goblet cells may also be present [10].

Primary cysts rarely impair vision. In contrast, implantations of the epithelium into the AC may progress over time and have a higher rate of recurrence and complications such as high intraocular pressure (IOP) and reduced best-corrected visual acuity (BCVA). Because secondary cysts can reach a significant size, corneal edema, uveitis, and secondary angle-closure glaucoma might also occur. Management options include Argon/Nd:YAG laser disruption of the cyst wall, fine-needle aspiration (sometimes coupled with an intracystic injection of absolute alcohol), or surgical excision [4,11,12,13].

We report a unique presentation and interaction occuring three years after clear corneal phacoemulsification in the left eye (OS) between an implantation iris cyst and the cornea in a patient with PBK. We searched the PubMed database for full-length English articles using the keywords “iris cyst”, “epithelial downgrowth”, “phacoemulsification”, “pseudophakic bullous keratopathy”, “cataract”, and, to our knowledge, this is the first reported occurrence of such an interaction. Given the limited number of reported iris cyst cases, documenting this unexpected interaction provides important data regarding their clinical behavior.

## 2. Case Presentation

**Figure 1:**
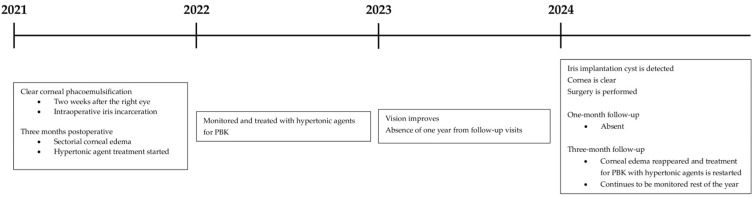
Timeline of the patient’s left eye (OS) clinical course over the years.

**Figure 2:**
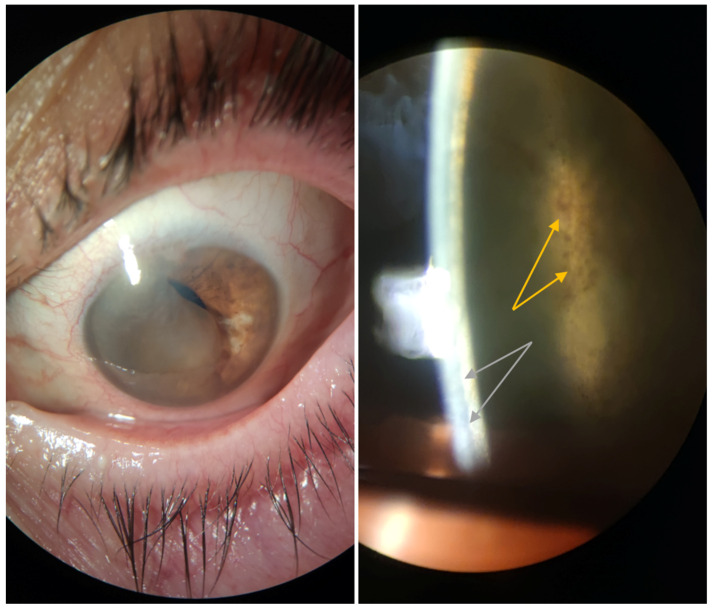
Anterior segment photograph of the OS at the slit-lamp, showing an iris cyst occupying 2/3 of the anterior chamber, obscuring the visual axis (**left panel**), displaying a probable solid component with pigment (yellow arrows) and in contact with the corneal endothelium (grey arrows) (**right panel**).

**Figure 3:**
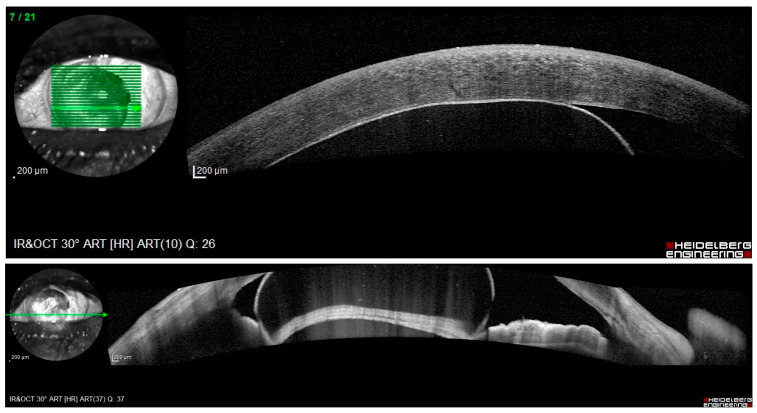
Anterior segment optical coherence tomography (OCT) using the OCT Spectralis showing contact of the iris cyst with the corneal endothelium (**upper panel**) and no extension of the lesion beyond the iris plane (**lower panel**).

A 75-year-old female with hypertonic agent-treated PBK in her OS since 2021 presented to our clinic in late 2024 after a one-year absence from follow-up. She reported a six-month history of decreased visual acuity following an initial improvement during the first half of her absence period, along with mild ocular discomfort (Figure 1). Family, medical, and trauma histories were unremarkable. Ocular history included bilateral clear corneal phacoemulsification with multifocal posterior chamber intraocular lens implantation in 2021: first in the right eye (OD), which was uneventful, and two weeks later in the OS, where iris incarceration occurred as the only intraoperative complication. Three months postoperatively, the patient developed sectorial corneal edema consistent with PBK. At presentation in 2024, BCVA was 1.0 OD and 2/50 OS, measured using the Snellen chart. Surprisingly, slit-lamp examination of the OS showed no signs of corneal edema or bullae, but revealed a cyst occupying 2/3 of the AC, with pigment on its surface and a possible solid component. This structure corresponded anatomically to the area where corneal edema had previously been observed and was located in proximity to the corneal endothelium (Figure 2). IOP was measured using Goldmann applanation tonometry, yielding values of 19 mmHg OD and 22 mmHg OS; OS fundoscopy was not possible, and while ultrasound biomicroscopy was unavailable at the time, anterior segment optical coherence tomography (OCT) (OCT Spectralis, Heidelberg Engineering, Heidelberg, Germany) indicated no lesion extension beyond the iris plane (Figure 3).

**Figure 4:**
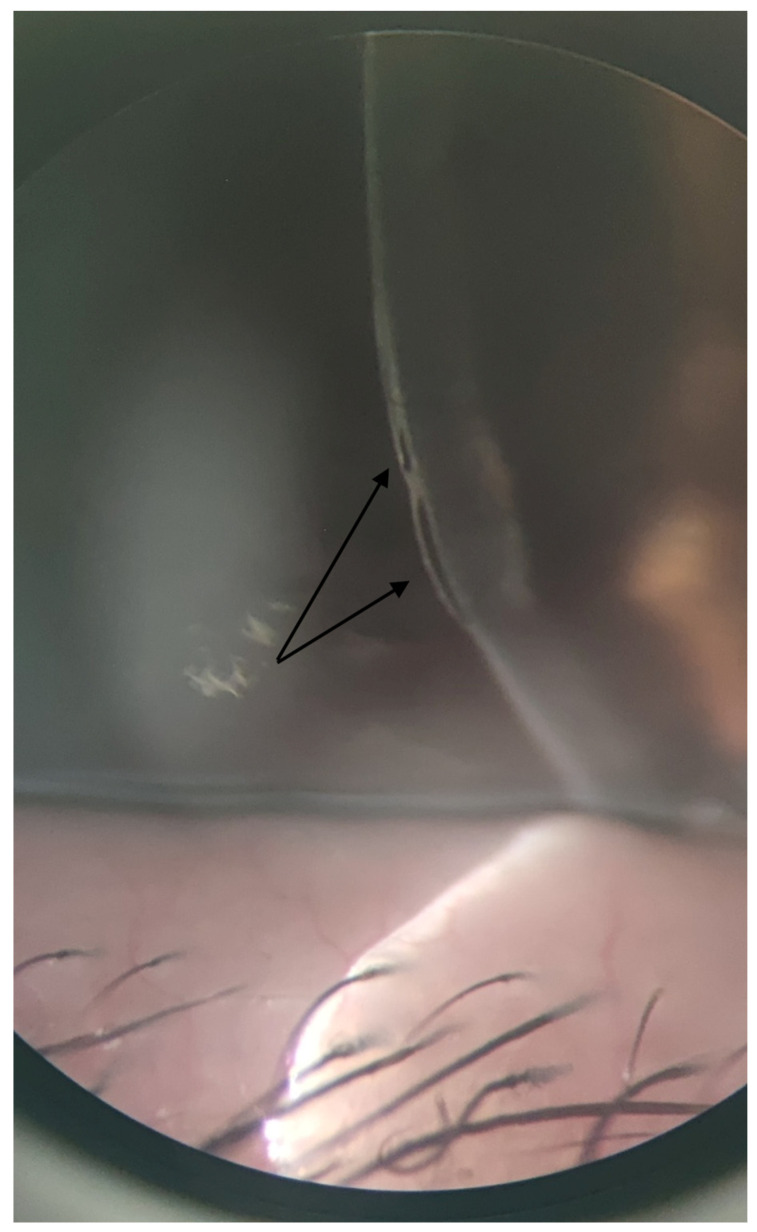
Anterior segment photograph of the OS at the slit-lamp at the three-month follow-up, showing the reappearance of corneal bullae (black arrows).

**Figure 5:**
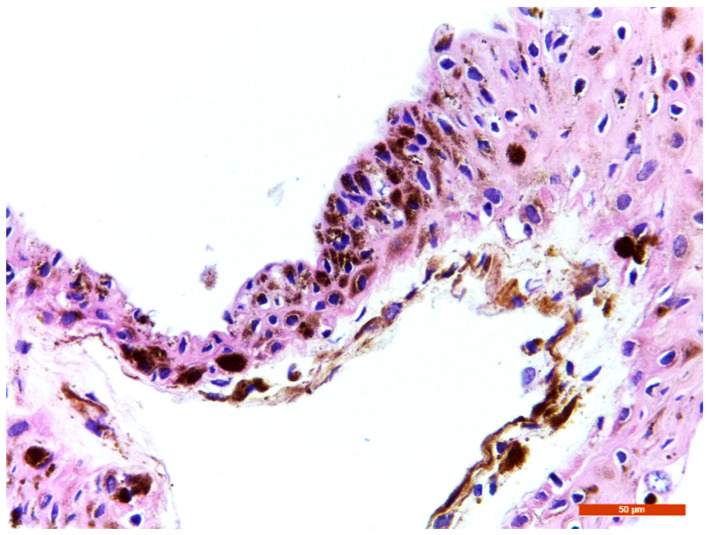
40× Haematoxylin–eosin staining of the iris cyst revealed a squamous non-keratinized stratified epithelium, with brown intracytoplasmic granules, without atypia. The underlying connective tissue also shows pigmentation.

A surgical approach was undertaken. The cyst was dissected using an ophthalmic viscoelastic device, and the specimen was sent for histopathological examination. On the third postoperative day, BCVA was 0.6, IOP was 18 mmHg, and the cornea was clear. The patient was subsequently discharged with a combination of antibiotic and corticosteroid eye drops for one month. She did not attend her one-month follow-up visit. At the three-month follow-up, she presented with pain, photophobia, decreased BCVA, and epithelial bullae once more (Figure 4). Treatment with a hypertonic agent was reinitiated, and she continues to be monitored. Histopathological analysis confirmed the diagnosis of an implantation iris cyst (Figure 5).

## 3. Discussion

Because iris cysts are uncommon, the literature consists mainly of a limited number of original research studies, and case reports [14,15,16,17,18], along with several review articles, most of which were published before 2000 [4,13,19,20,21,22,23]. Research on this topic is limited due to the challenge of assembling a sufficiently large group of patients with iris cysts. Many patients with this condition do not seek ophthalmologic evaluation, and among those who do, iris cysts are often detected incidentally during examination for unrelated reasons.

One group recently studied 39 patients, examining the morphological features, location, and long-term complication risk of primary and secondary cysts, and found a higher prevalence in women and a mean age at diagnosis of 41.6 years [11]. Similar data were reported by Köse et al. [24] and Marigo et al. [13]. While age significantly helps differentiate iris cysts from melanomas, the patient’s age in our case made diagnosis particularly difficult. The horizontal size of the cysts is positively correlated with age and negatively correlated with visual acuity [11]. Paradoxically, in our patient, the cyst’s growth initially improved vision until it reached a point at which it began to obstruct the visual axis, elevate IOP, and cause pain.

Melanomas are more frequently encountered in men. Given that most cases of ED occur within the first year after intraocular surgery, the lesion’s size, solid component, pigmentation, and the patient’s age raised suspicions for an iris melanoma, either developing from a benign implantation cyst or representing a cystic melanoma. McGarth et al. reported the case of cavitary iris melanoma in a 34-year-old male patient presenting with a pigmented cystic lesion [25]. Similar to cysts, iris and ciliary body melanomas are located in the lower quadrants in over 80% of cases; therefore, lesion location cannot serve as a differentiating clinical feature for iris cysts [3]. However, no cytological atypia was identified in our case, as shown in Figure 5. Histopathological examination confirmed that the lesion was an iris implantation cyst, a subtype of secondary iris cyst resulting from the implantation of epithelial cells into the AC, most likely due to incomplete wound closure and iris incarceration during cataract surgery. Clinically, the cyst appeared several months after surgery, enlarged progressively, and eventually came into contact with the posterior corneal surface.

Sutureless phacoemulsification has become the norm, with very few cases of ED reported over the past 30 years, as tissue manipulation has been minimized. Introduction of epithelial cells into the AC can occur if surgical instruments carry them over during procedures. Despite advances in technologies like femtosecond laser-assisted cataract surgery, where corneal incisions are made with a laser instead of manual tools, iris cysts can still develop, as Wu et al. reported [14]. These laser-created incisions have advantages such as lower rates of endothelial gap formation, misalignment, and Descemet membrane detachment, as well as smaller wound size and higher precision, however they do not eliminate the risk of iris cysts.

The incidence of both complications (secondary iris cyst and PBK) is low (<0.1%). Our extensive literature review suggests this is the first reported case in which these two conditions co-develop within the same eye and, more importantly, display a unique interaction. Unexpectedly, during the period of maximal cyst size, the patient’s corneal edema improved, suggesting that the cyst may have acted as a mechanical barrier, temporarily limiting aqueous humor influx into the cornea. As previously mentioned, the edema initially affected a sector of the cornea, correlating with the area of cyst contact, supporting the spatial relationship hypothesis.

Interestingly, when questioned about her one-year absence, the patient reported that her pain subsided as her vision improved, which led her to discontinue treatment and not return for follow-up, believing her condition had been cured. We hypothesize a mechanical barrier effect, where the cyst physically covered the endothelial defect, preventing the influx of aqueous humor into the cornea, and leading to the observed improvement in both pain and visual acuity. This action resembles EyeYon Medical’s EndoArt^®^ Corneal Artificial Endothelial Layer, an artificial water-impermeable device designed to adhere to the posterior portion of the cornea and treat PBK.

There are several other factors and limitations in our case that need to be discussed. At the time of the initial 2021 presentation, the case was considered routine, and slit-lamp photographs were not obtained. Given the long intervals between follow-up visits, it cannot be excluded that the iris cyst was present at an earlier stage but remained clinically unapparent or undetected. A small or preclinical lesion may have been overlooked before it subsequently enlarged and became clearly visible on slit-lamp examination.

Another limitation is that specular microscopy was unavailable at initial examination or at any follow-up, and endothelial cell count could not be measured. Preoperative findings and the fellow eye did not show features suggestive of Fuchs endothelial dystrophy (no guttae, no morning blur, no bilateral involvement). The iris implantation cyst in this case reached a significant size within the AC, coming into proximity with the posterior corneal surface. Endothelial status was assessed clinically using slit-lamp examination, and endothelial cell loss secondary to iris cyst-endothelium contact cannot be excluded.

Several additional mechanisms may have contributed to the patient’s improved vision and pain relief, and consequently reduced her adherence to follow-up visits. Neuroadaptation may have played a role, as the visual system had been exposed to chronically blurred input due to PBK. The apparent improvement reported by the patient may be explained by cortical neuroadaptation and functional monocular reliance. As the visual system increasingly suppressed input from the affected eye and relied on the fellow eye, the perceived visual impairment diminished. Another possible contributing factor could have been the cyst’s pinhole-like effect—partially obstructing the pupil and improving vision until it expanded and occupied the visual axis, decreasing vision again and ultimately forcing the patient to seek medical care.

Additionally, the proposed mechanical barrier mechanism remains speculative, as it cannot be directly proven from observational data alone. Other variables also need to be taken into consideration. Despite the anatomical improvement, visual acuity was low at the initial 2024 presentation (2/50). This is most likely due to the cyst occupying the visual axis, as after its removal, visual acuity increased significantly, but did not reach its full potential. Thus, additional factors such as underlying structural corneal changes due to the previous chronic edema or endothelial changes must also be considered, as large anterior cysts are known to pose a risk to endothelial cells. Although this mechanism is theoretical, the clinical course, temporal correlation, and anatomical relationship indicate a temporary functional interaction, suggesting more than a coincidental coexistence.

## 4. Conclusions

In conclusion, this case demonstrates the development of a histopathologically confirmed iris implantation cyst following cataract surgery in a patient with PBK. The cyst’s enlargement appeared to temporarily improve corneal clarity, potentially through a mechanical barrier effect that limits aqueous humor influx. While the observations suggest a potential functional interaction between the cyst and the cornea, the proposed mechanism remains speculative. Nevertheless, the rarity of these serious complications, combined with this unique interplay, highlights the complex relationship between iris implantation cysts, corneal physiology, and the dynamics of ocular surgery, emphasizing the importance of careful follow-up and histopathologic confirmation for accurate diagnosis. This case serves as a reminder that postoperative complications can arise at any time, and surgeons must be prepared to identify and manage them accordingly. Close communication with the pathologist is also essential for achieving the best possible outcome for the patient.

## Data Availability

The original contributions presented in this study are included in the article. Further inquiries can be directed to the corresponding author.

## Abbreviations

The following abbreviations are used in this manuscript:

PBK	Pseudophakic bullous keratopathy
ED	Epithelial downgrowth
AC	Anterior chamber
IOP	Intraocular pressure
BCVA	Best-corrected visual acuity
OS	Left eye
OD	Right eye
OCT	Optical coherence tomography
